# Suramin Exerts an Ameliorative Effect on Acetic Acid-Induced Acute Colitis in Rats by Demonstrating Potent Antioxidant and Anti-Inflammatory Properties

**DOI:** 10.3390/medicina61050829

**Published:** 2025-04-30

**Authors:** Gulcin Ercan, Hatice Aygün, Ahmet Akbaş, Osman Sezer Çınaroğlu, Oytun Erbas

**Affiliations:** 1 Department of General Surgery, Sultan 2. Abdulhamid Han Educational and Research Hospital, Istanbul Provincial Directorate of Health, Istanbul 34865, Turkey; ghepgul@hotmail.com; 2 Faculty Medicine, Department of Physiology, Tokat Gaziosmanpaşa University, Tokat 60250, Turkey; 3 Department of General Surgery, Faculty of Medicine, Karadeniz Technical University, Trabzon 61080, Turkey; draakbas@hotmail.com; 4 Department of Emergency Medicine, Faculty of Medicine, Izmir Katip Çelebi University, Izmir 35620, Turkey; drsezer@hotmail.com; 5 Faculty of Medicine, BAMER, Biruni University, Istanbul 34015, Türkiye; oytunerbas2012@gmail.com

**Keywords:** suramin, anti-inflammatory activity, antioxidant potential, acute experimental colitis

## Abstract

*Background and Objectives*: The purpose of this study was to evaluate potential protective effects of suramin on inflammation, oxidative stress, and histopathological damage a rat model of acute colitis created with acetic acid. *Materials and Methods*: Wistar albino (male) rats were randomly assigned to three groups: control (n = 10), colitis + saline (n = 10), and colitis + suramin (n = 10). Rectal instillation of 4% acetic acid was used to induce acute colitis. Suramin (10 mg/kg/day) or saline was administered intraperitoneally for 15 days. Plasma concentrations of pentraxin 3 (PTX3), tumor necrosis factor-alpha (TNF-α), neutrophil extracellular traps (NETs), and malondialdehyde (MDA) were determined using enzyme-linked immunosorbent assay (ELISA) and spectrophotometric methods. In addition, vascular endothelial growth factor (VEGF) and TNF-α levels in colonic tissue were also measured. Histopathological evaluations were conducted using hematoxylin and eosin staining. *Results:* Significant increases in plasma and tissue inflammatory markers, oxidative stress parameters, and histopathological scores were observed when compared to control group; values were higher in colitis group. Suramin treatment significantly reduced plasma PTX3, TNF-α, NETs, and MDA levels, and colonic TNF-α and VEGF concentrations compared to the untreated colitis group. Histological analysis showed reduced epithelial injury and leukocyte presence in rats receiving suramin. *Conclusions*: Our findings demonstrate that suramin significantly attenuates inflammatory and oxidative damage in an experimental model of acute colitis. These results suggest that suramin may possess therapeutic potential in intestinal inflammation; however, this effect requires further support through advanced experimental and clinical studies.

## 1. Introduction

Acute colitis (AC) refers to a sudden inflammatory episode in the colon, which may occur independently or as part of a chronic relapsing condition such as ulcerative colitis (UC), significantly impairing quality of life [1,2]. It affects approximately 25 out of 10,000 individuals, with its prevalence increasing globally [3,4]. Recent reports emphasize the growing burden of inflammatory bowel diseases (IBD), including Crohn’s disease (CD) and UC, particularly among young women in both developed and developing countries [4,5]. Environmental factors such as dietary imbalances, antibiotic use, and psychosocial stress have also been implicated in AC onset and relapse, highlighting its multifactorial etiology [6]. Despite the availability of pharmacological treatments, current therapies are often limited by suboptimal efficacy and adverse effects, underscoring the need for safer and more effective alternatives [7,8]. In this context, drug repurposing has emerged as a promising strategy, particularly for well-characterized compounds with known safety profiles [9].

Earlier research indicates that several factors have a role in the development of AC, including genetic susceptibility, autoimmune disorders, and imbalances in gut microbiota [10,11]. The primary pathological alteration observed in AC is an exaggerated immune response resulting from elevated levels of proinflammatory cytokines, notably interleukin-6 (IL-6), interleukin-1 beta (IL-1β), and tumor necrosis factor-alpha (TNF-α). Activation of proinflammatory cytokines leads to increased permeability of epithelial cells, immune cell infiltration (neutrophils, T helper cells and macrophages), and thus mucosal inflammation [12]. Simultaneously, oxidative stress impairs epithelial integrity through increased reactive oxygen species (ROS) and exacerbates mucosal damage by worsening inflammation [13]. Targeting oxidative stress may reduce inflammation and support epithelial barrier integrity, which is critical in gastrointestinal diseases driven by redox imbalance [14].

Suramin is one of the oldest FDA-approved drugs widely used in the treatment of onchocerciasis and African trypanosomiasis [15]. It exhibits a wide range of biological effects including antiviral activity, anti-inflammatory, anticancer, and antiproliferative. Suramin has been shown to reduce inflammation in many different experimental studies. For example, one study showed that suramin reduced central nervous system (CNS) inflammation in a rat brain trauma model [16]. Another study showed that suramin inhibited inflammation in a mouse model of atopic dermatitis [17]. Findings from prior studies suggest that suramin attenuates lung tissue inflammation through suppression of lipopolysaccharide (LPS)-induced TNF-α and IL-6.

Based on the literature reviewed, suramin appears to have potential anti-inflammatory effects in models of intestinal inflammation. However, its specific efficacy in acute colitis has not been clearly established. In this study, we used an acetic acid-induced acute colitis model to mimic acute intestinal inflammation with features resembling ulcerative colitis. We aimed to evaluate the histopathological and biochemical effects of suramin in the AA-induced acute colitis model.

## 2. Materials and Methods

### 2.1. Animals

All procedures complied with the Guide for the Care and Use of Laboratory Animals (NIH, Bethesda, MD, USA). The protocol received ethical approval by the Animal Ethics Committee (Approval No: 0824073823; Date: 12 April 2022). Wistar rats were obtained from the university’s Laboratory for Experimental Animals. Animals were housed in pairs in steel cages under controlled conditions (22 ± 2 °C; 12-h light/dark cycle). The sample size (n = 10 per group) was determined based on previous studies using the same colitis model [18,19], in accordance with ethical 3R principles to minimize animal use while ensuring reliable outcomes.

Thirty adult male Wistar albino rats (8–10 weeks old, 200–250 g) were used. Rats were randomly assigned to groups using a computer-generated number sequence.

### 2.2. Experimental Model of Acute Colitis

In this study, an experimental model of acute colitis was utilized to replicate acute intestinal inflammation resembling some features of ulcerative colitis, rather than modeling chronic inflammatory bowel disease.

Acute colitis was experimentally induced in 20 rats via rectal instillation of acetic acid (AA, 1 mL 4%) solution (Merck Sigma-Aldrich, St. Louis, MO, USA) under ether anesthesia (ketasol/xylazine −100/50 mg/kg). A soft catheter was inserted 6 cm into the rectum, and AA was slowly instilled, followed by 1 mL of air to ensure proper distribution and prevent leakage. Ten rats served as the control group and received no intervention.

**Figure 1:**
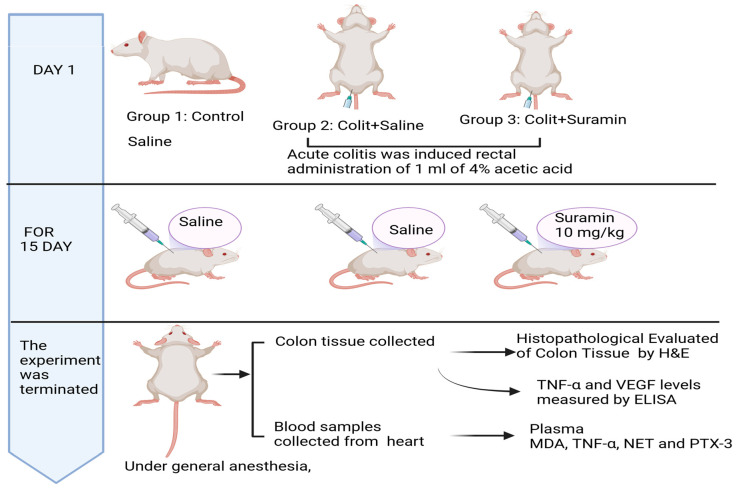
Experimental timeline.

The 20 colitis-induced rats were randomly divided to two groups. Group 1 received 0.9% NaCl (1 mL/kg/day, intraperitoneally), and Group 2 received suramin (10 mg/kg/day, intraperitoneally; Merck Sigma-Aldrich, USA). Treatments were carried out over 15 days. Upon completion of the study, the rats were euthanized. Blood samples were obtained through cardiac puncture for biochemical analysis, and colon tissues were harvested for tissue examination and biochemical assessment (Figure 1).

### 2.3. Histopathological Evaluation of Colon Tissue

Colon samples were fixed in formalin, sectioned at 4 μm, and stained with hematoxylin and eosin. Images were captured using an Olympus C-5050 digital camera attached to an Olympus BX51 microscope. Histopathological scoring was performed according to the criteria of MacPherson and Pfeiffer (1976) [20]:

0: Intact epithelium; no leukocyte infiltration or hemorrhage
1: <25% epithelial damage with focal leukocyte infiltration and hemorrhage
2: 25% epithelial disruption with focal infiltrates and hemorrhage
3: 50% epithelial damage with widespread leukocyte infiltration and hemorrhage
4: >50% epithelial damage with extensive infiltration and hemorrhage

### 2.4. Measurement of Plasma TNF-α and NETs Levels

Plasma concentrations of tumor necrosis factor-alpha (TNF-α) and neutrophil extracellular traps (NETs) were measured using commercially available enzyme-linked immunosorbent assay (ELISA) kits according to the manufacturer’s protocols. Prior to the assay, plasma samples were centrifuged at 3000 rpm for 10 min at 4 °C to remove any particulate matter. Supernatants were collected and stored at −80 °C until analysis.

Samples were diluted 1:2 with the assay buffer provided in the kits and each sample was analyzed in duplicate, according to the manufacturer’s instructions. Briefly, 100 µL of each standard, blank, and diluted sample was added to the wells of pre-coated microplates and incubated for the recommended duration at room temperature. Following incubation, wells were washed thoroughly, and detection antibodies were added. After final substrate incubation, optical density was measured at 450 nm using a microplate reader (BioTek ELx808; BioTek Instruments, Inc., Winooski, VT, USA). Standard curves were generated using known concentrations, and sample values were calculated accordingly. TNF-α levels were expressed in pg/mL, and NETs concentrations were presented in ng/mL.

### 2.5. Measurement of Plasma PTX3

Plasma concentrations of pentraxin 3 (PTX3) were measured using a commercially available sandwich ELISA kit (USCN Life Science Inc., Wuhan, China). Blood samples were collected into EDTA-containing tubes and centrifuged at 3000 rpm for 10 min at 4 °C. Plasma was separated, aliquoted, and stored at −80 °C until analysis to preserve protein stability.

For the assay, 100 μL of each plasma sample was analyzed in duplicate according to the manufacturer’s instructions. Absorbance was measured at 450 nm using a microplate reader (BioTek ELx808/USA). PTX3 concentrations were calculated based on the standard curve generated from known concentrations and expressed in ng/mL.

### 2.6. Detection of TNF-α and VEGF in Colon Tissue

Frozen colon samples were homogenized in PBS buffer (pH 7.2) containing PMSF, Pepstatin A, aprotinin, and leupeptin. Homogenates were centrifuged at 12,000 rpm for 20 min at 4 °C, and supernatants were collected. Protein levels were quantified using the Bradford method TNF-α and vascular endothelial growth factor (VEGF) levels were measured using ELISA kits (rat-specific), In line with the instructions supplied by the manufacturer. Absorbance values were measured at 450 nm. The results are presented in pg per mg of tissue.

### 2.7. Plasma Lipid Peroxidation Measurement

Plasma malondialdehyde (MDA) levels, an indicator of lipid peroxidation, were determined using the thiobarbituric acid reactive substances (TBARS) assay. The method is based on the formation of an MDA–TBA adduct, which produces a pink chromogen measurable by spectrophotometry. Plasma samples were mixed with equal volumes of TBA reagent (0.67% TBA in 20% acetic acid, pH 3.5) and heated in a boiling water bath at 100 °C for 60 min. After cooling to room temperature, the samples were centrifuged at 3000 rpm for 20 min. The absorbance of the resulting supernatant was measured at 535 nm using a spectrophotometer (BioTek/BioTek Instruments, Inc., Winooski, VT, USA). Tetraethoxypropane was used to generate the standard curve, and MDA concentrations were expressed as nmol/mL.

### 2.8. Statistical Analysis

Prior to statistical comparisons, the distribution of each dataset was assessed using the Kolmogorov–Smirnov test. The histopathological score, being ordinal in nature, did not follow a normal distribution; therefore, statistical comparisons were performed using the non-parametric Mann–Whitney U test, although data were expressed as mean ± SEM for clarity. Among biochemical parameters, plasma TNF-α, NETs, PTX3, MDA levels and colonic TNF-α, VEGF levels were normally distributed (*p* > 0.05), thus analyzed using one-way ANOVA followed by Tukey’s post hoc test. All results are presented as mean ± standard error of the mean (SEM). Statistical significance was set at *p* < 0.01, while *p* < 0.001 was considered highly significant. All statistical analyses were performed using SPSS version 15.0 (SPSS Inc., Chicago, IL, USA) and GraphPad Prism version 10.0 (GraphPad Software, San Diego, CA, USA). All graphs were generated using GraphPad Prism version 10.0.

## 3. Results

### 3.1. Histopathological Evaluation

**Figure 2:**
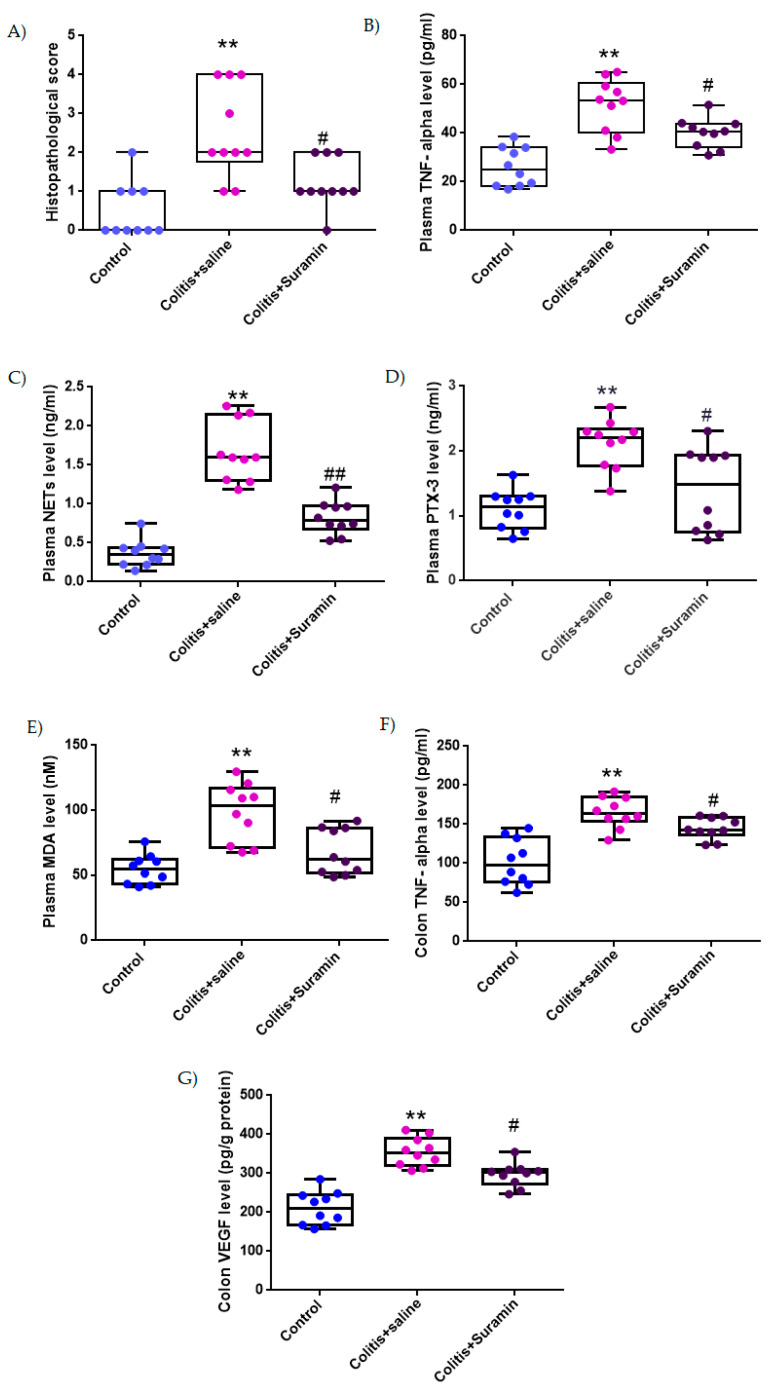
Box plots representing histopathological scores (**A**), plasma levels of TNF-α (**B**), NETs (**C**), PTX3 (**D**), and MDA (**E**), and colonic levels of TNF-α (**F**) and VEGF (**G**) in control, colitis + saline, and colitis + suramin-treated groups. Data are presented as mean ± SEM. Statistical significance was evaluated by one-way ANOVA followed by Tukey’s post hoc test, except for histopathological score, which were analyzed using Kruskal–Wallis test followed by Mann–Whitney U test. ** *p* < 0.01 vs. control group; # *p* < 0.01, ## *p* < 0.001 vs. colitis + saline group.

**Figure 3:**
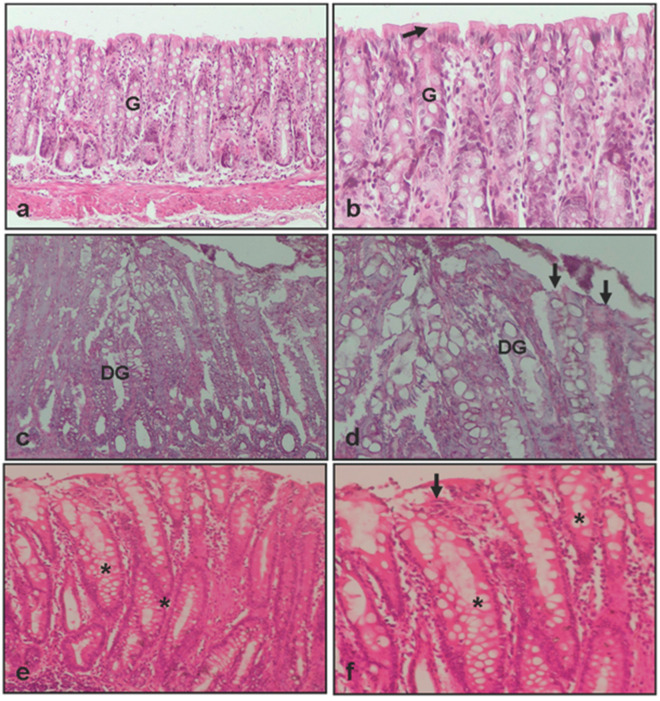
Light microscopical images of colon from experimental groups. (**a**,**b**) Colon tissue of control group, showing normal epithelium (arrow) and gland (G); (**c**,**d**) colon tissue of colitis +0.9% NaCl group, showing disrupted epithelium (arrow) and degenerated gland (DG); (**e**,**f**) colon tissue of colitis +10 mg/kg suramin group, showing healed epithelium (arrow) and gland (*)*:* Hematoxylin and eosin, ×0 and ×20.

Although the histopathological scoring system is ordinal by nature, descriptive statistics were expressed as mean ± SEM to facilitate clarity and comparison across groups, especially given the relatively symmetrical distribution of the data. However, in accordance with the ordinal characteristics of the data, all statistical comparisons were performed using the non-parametric Mann–Whitney U test. The control group exhibited a mean score of 0.5 ± 0.2, while the colitis group treated with saline showed significantly increased damage (2.5 ± 0.4; ** *p* < 0.001). Notably, suramin treatment significantly reduced the histopathological score to 1.2 ± 0.2 compared to the colitis group (# *p* < 0.01), indicating a protective effect. These differences were confirmed by Mann–Whitney U test (Figure 2 and Figure 3 and Table 1).

### 3.2. Plasma TNF-α Concentrations

Plasma TNF-α, a key pro-inflammatory cytokine, was measured at 26.1 ± 2.5 pg/mL in the control group. In the colitis + NaCl group, levels significantly increased to 51.6 ± 3.4 pg/mL (** *p* < 0.001), reflecting systemic inflammation. Treatment with suramin resulted in a marked decrease in TNF-α levels (40.1 ± 1.9 pg/mL), demonstrating significant attenuation of inflammatory response compared to colitis group (# *p* < 0.01), (Figure 2 and Table 1).

### 3.3. Plasma NETs Levels

NET levels in plasma were found to be 0.36 ± 0.05 ng/mL in healthy controls. In the colitis + NaCl group, NETs levels increased substantially to 1.67 ± 1.2 ng/mL (** *p* < 0.001), indicating excessive neutrophil activation. Suramin treatment led to a significant reduction in NET formation (0.82 ± 0.06 ng/mL), when compared to untreated colitis group (## *p* < 0.001), (Figure 2 and Table 1).

### 3.4. Plasma PTX3 Concentrations

Plasma PTX3, an acute-phase protein associated with tissue damage and inflammation, was recorded as 1.1 ± 0.09 ng/mL in the control group. In colitis + NaCl group, levels increased significantly to 2.1 ± 0.1 ng/mL (** *p* < 0.01). Suramin treatment reduced PTX3 concentrations to 1.4 ± 0.2 ng/mL, with a significant difference from the colitis group (# *p* < 0.01), suggesting reduced tissue stress (Figure 2 and Table 1).

### 3.5. Plasma MDA Levels

MDA is recognized as an indicator of lipid peroxidation and oxidative damage was measured at 54.6 ± 3.5 nM in control group. MDA levels rose significantly to 98.2 ± 7.1 nM in the colitis + NaCl group (** *p* < 0.001), indicating oxidative damage. Suramin administration significantly lowered plasma MDA levels to 67.8 ± 5.5 nM compared to colitis group (# *p* < 0.01), demonstrating its antioxidant potential (Figure 2 and Table 1).

### 3.6. Colonic TNF-α Levels

Colonic tissue TNF-α levels were 101.7 ± 9.3 pg/mg in control group. A significant elevation was observed in colitis + NaCl group (165.08 ± 6.2 pg/mg, ** *p* < 0.01), highlighting local inflammatory activation. In contrast, the colitis + suramin group exhibited significantly reduced colonic TNF-α levels (144.6 ± 4.3 pg/mg) compared to the colitis group (# *p* < 0.01), indicating effective suppression of local inflammation (Figure 2 and Table 1).

### 3.7. Colonic VEGF Levels

VEGF levels in colonic tissue, reflecting angiogenic activity and tissue remodeling, were 210.6 ± 13.5 pg/g protein in the control group. VEGF levels were markedly elevated in the colitis + NaCl group (354.8 ± 11.6 pg/g protein, ** *p* < 0.001). In the suramin group, VEGF levels significantly decreased to 295.9 ± 9.7 pg/g protein compared to colitis group (# *p* < 0.01), suggesting attenuation of pathological angiogenesis (Figure 2 and Table 1).

## 4. Discussion

This study investigates the protective effect of suramin against acetic acid-induced colitis in a rat model. The preventive effect of suramin was confirmed by biochemical and histological evaluation. Fifteen-day treatment with suramin markedly decreased colonic damaged caused by and prevented inflammatory response and oxidative stress.

AA-induced colitis in rats has been widely used as a clinical model AC [21]. The model mimics the mechanisms involved in the onset of human AC, such as gastrointestinal inflammation, villus atrophy, and ulceration [22,23]. Similarly, the results of this study show that AA-induced AC causes tissue damage and deterioration in biochemical parameters. Suramin administration decreased the severity of colitis; this effect was supported by decreased serum TNF-α, NETs, PTX3, and MDA levels and suppressed TNF-α and VEGF levels in colonic tissue. In addition, histopathological evaluation showed that suramin treatment attenuated epithelial damage and leukocyte infiltration in colitis rats.

Although the mechanism that causes ulcerative colitis is still not fully known, it is accepted that TNF-α and oxidative stress triggered by them play a part in the onset and progression of the disease in a complex interaction [22,23,24]. Recent reviews have confirmed that persistent TNF-α overexpression not only triggers but also maintains chronic inflammation in ulcerative colitis by disrupting epithelial barrier integrity and amplifying mucosal immune responses [10,25]. Numerous studies have demonstrated elevated TNF-α expression in both patients with acute colitis and in various experimental models of the disease [26]. Increased TNF-α triggers neutrophils and macrophages to phagocytose and produce effector mediators. Furthermore, studies have reported that TNF-α promotes intestinal inflammation, and its upregulation is associated with increased ulcerative colitis (UC) severity [27]. In a study, it was reported that TNF-α level and neutrophil infiltration increased. Cell necrosis and oedema were observed in the colonic mucosa of rats with AA-induced colitis [28]. This study found elevated TNF-α levels in the colon and plasma of AA-treated rats, aligning with prior research.

Under normal conditions, various signals such as viruses, bacteria, parasites, yeasts, and cytokines trigger the formation of NETs from neutrophils and are necessary for their elimination, but when they are excessively secreted they cause cell damage [29,30]. A growing body of research have reported that NET-associated proteins are increased in plasma and colonic cells of AC patients and an experimental model of AC [30,31,32,33]. A clinical study revealed that the NET level in colonic mucosa in patients with AC was approximately 42.2-fold higher than in normal colons [34]. Additionally, previous studies indicated that NET plasma levels are remarkably elevated in patients with AC [35]. In a recent study by Li and colleagues, it was shown that plasma levels of NET were increased in mice with dextran sulphate sodium (DSS)-induced colitis model [36]. In agreement with prior research, the present study observed that the level of NET was increased in the plasma of rats with colitis.

Higher concentrations of TNF-α exacerbate the severity of inflammation of the colonic mucosa both directly and indirectly by stimulating neutrophil activation and NET formation [37]. TNF-α initiates an intracellular signaling cascade in neutrophils, triggering the shedding of chromatin traps and resulting in NET formation. Previous studies have shown that over-activation of NETs may amplify the inflammation-mediated response, leading to cell damage [38]. Long et al. [38] reported that excessive NET accumulation exacerbates inflammation of the AC disrupting the structure of the colonic mucosal barrier. Moreover, previously documented indicated that NETs trigger the overproduction of pro-inflammatory cytokines from immune cells, thus promoting inflammation.

Suramin may reduce colitis severity by lowering TNF-α and NET levels. Firstly, in previous studies, it was reported that suramin disrupts the active trimeric structure of TNF-α and inhibits its biological activity by preventing binding to its receptor [39]. Similarly, Mancini et al. [40] showed that suramin can suppress TNF-α/TNF-α ligand–receptor interaction in a dosage-related manner. TNF-α inhibition has long been known to be a critical treatment option for AC patients. For this reason, anti-TNF-α therapy is widely used in the clinical management of inflammatory bowel diseases, including ulcerative colitis and Crohn’s disease [41]. In the light of our results, suramin showed anti-inflammatory effect in AA-induced AC model.

Secondarily, suramin directly reduces neutrophil-induced NET damage. Positively charged histone proteins released from NETs can cause toxicity in cells and trigger inflammatory responses [30]. Thanks to its polyanionic structure, suramin can neutralize these free positive histones by binding to them. Indeed, one study showed that suramin markedly reduced histone-induced neutrophil upregulation in lung endothelial cells [42].

Villalba et al. [42] reported that suramin suppresses endothelial activation by neutralizing extracellular histones, supporting its broader anti-inflammatory potential, consistent with our findings. Similarly, Long et al. [38] identified NETs as key contributors to colitis progression, reinforcing the NET-targeting effect of suramin observed in our study. Our findings, in line with previous research, indicate that suramin may protect the colonic mucosa by simultaneously suppressing TNF-α and NET-associated inflammation.

In the AA-induced AC model, suramin administration may suppress TNF-α release and NET-forming effect of TNF-α. It may also prevent cellular damage in the colon mucosa by inactivating the harmful products of formed NETs.

Pentraxin 3 (PTX3) acts as a serum acute-phase protein, upregulated rapidly during sepsis, endotoxic shock, and infectious conditions [43]. Kato et al. (2008) demonstrated that plasma PTX3 levels were significantly elevated in patients with active inflammatory bowel disease compared to those with inactive disease and healthy controls [44]. Previous studies have shown that PTX3 is secreted in response to pro-inflammatory cytokines such as TNF-α and IL-1β [45]. Indeed, PTX3 itself may be a factor that increases inflammation; in a study in mice, it was shown that in the absence of PTX3, NF-κB activation and TNF-α production decreased while tissue damage was attenuated [46]. The PTX3-lowering effect of suramin observed here aligns with previous findings and may reflect a convergence of cytokine modulation and neutrophil activity control.

It has been reported that PTX3 is stored in neutrophil granules and approximately 25% is released into the external environment when stimulated [47]. In a cell culture study, it was reported that PTX3 expression was observed in stimulated human neutrophils [48]. Previously studies have reported that PTX3 can be partially localized in NETs [43,47]. Therefore, PTX3 expression can be considered as an indicator of severe neutrophil activation. Suramin may also restrict the main source of PTX3 by reducing neutrophil infiltration, as well as inhibiting NET and PTX3 production by suppressing TNF-α.

Activated neutrophils and immune cells increase reactive oxygen species, leading to lipid peroxidation and malondialdehyde (MDA) formation [49,50]. MDA indicates oxidative tissue damage. Oxidative stress and ROS are key factors in AC development. Additionally, ROS pathways may upregulate PTX3 and NET expression, and elevated TNF-α levels have been shown to enhance PTX3 production and may also promote NET formation, potentially contributing to inflammation in AUC [51,52].

VEGF plays a central role in angiogenesis, a process essential for tissue repair but also contributes to inflammation. During active inflammation, macrophage-derived TNF-α stimulates VEGF release, increasing vascular permeability. In experimental colitis models, elevated VEGF levels promote excessive leukocyte infiltration and exacerbate mucosal injury [53]. Tolstanova et al. [54,55] highlighted VEGF upregulation in active lesions, along with increased vascular leakage. Moreover, administering anti-VEGF antibodies in colitis models reduced colonic permeability, inflammatory infiltration, and mucosal damage.

Consistent with these findings, Betto et al. [53] demonstrated that inhibition of VEGF receptor signaling promotes mucosal healing in dextran sulfate sodium (DSS)-induced colitis. Our results similarly showed that suramin treatment was associated with decreased VEGF levels and reduced mucosal injury. Taken together, these findings suggest that suramin’s VEGF-modulatory effects may hold therapeutic value by limiting angiogenesis and supporting epithelial restoration in colitis.

## 5. Conclusions

The findings of this study suggest that suramin exerts a protective effect against acetic acid-induced acute colitis by modulating key inflammatory and oxidative pathways. The significant downregulation of pro-inflammatory cytokines (TNF-α), neutrophil-derived inflammatory mediators (NETs and PTX3), and oxidative stress marker (MDA) in plasma, as well as the marked reduction in tissue TNF-α and VEGF levels, highlight its potent anti-inflammatory, antioxidant, and anti-angiogenic properties. By limiting epithelial injury and immune cell accumulation, suramin contributed to the preservation of structural organization within the colonic mucosa.

Collectively, these results provide a solid experimental foundation indicating that suramin may effectively alleviate acute colitis by suppressing the TNF-α/NET/PTX3 axis and reducing ROS-mediated tissue injury. Its additional capacity to limit pathological angiogenesis through VEGF modulation further strengthens its therapeutic promise. To our knowledge, this is one of the first studies to explore the role of suramin in regulating NETs and VEGF in colitis, thus contributing new mechanistic insights to the field. Further research should explore the optimal dosage, assess long-term safety, and confirm suramin’s effects in chronic and clinical models of ulcerative colitis.

## 6. Limitations

As this study represents the first evaluation of suramin in experimental colitis, a positive control group was not included in order to minimize animal use; however, future studies will incorporate dose–response and comparative evaluations with standard therapies.

## Figures and Tables

**Table 1:** Histopathological and biochemical findings of experimental groups of colitis.

	Control Group	Colitis + Saline	Colitis + Suramin
Histopathological score	0.5 ± 0.2	2.5 ± 0.4 **	1.2 ± 0.2 #
Plasma TNF-α level (pg/mL)	26.1 ± 2.5	51.6 ± 3.4 **	40.1 ± 1.9 #
Plasma NETs level (ng/mL)	0.36 ± 0.05	1.67 ± 0.12 **	0.82 ± 0.06 ##
Plasma PTX3 level (ng/mL)	1.1 ± 0.09	2.1 ± 0.1 **	1.4 ± 0.2 #
Plasma MDA level (nM)	54.6 ± 3.5	98.2 ± 7.1 **	67.8 ± 5.5 #
Colon TNF-α level (pg/mg tissue)	101.7 ± 9.3	165.08 ± 6.2 **	144.6 ± 4.3 #
Colon VEGF level (pg/g protein)	210.6 ± 13.5	354.8 ± 11.6 **	295.9 ± 9.7 #

Results are presented as mean ±SEM. Statistical analyses were performed using one-way ANOVA followed by Tukey’s post hoc test, except for histopathological score, which were analyzed using Kruskal–Wallis test followed by Mann–Whitney U test. ** *p* < 0.001 vs. control group; # *p* < 0.01, ## *p* < 0.001 vs. colitis + saline group. TNF-α: tumor necrosis factor-alpha; NET: neutrophil extracellular traps; PTX3: pentraxin 3; MDA: malondialdehyde; VEGF: vascular endothelial growth factor.

## Data Availability

Data is available on request due to ethical/privacy restrictions.
